# A Pediatric Case of Narcolepsy Presenting as Syncope

**DOI:** 10.7759/cureus.3801

**Published:** 2018-12-31

**Authors:** Jeffrey A Miskoff, Moiuz Chaudhri

**Affiliations:** 1 Internal Medicine, Jersey Shore University Medical Center, Neptune City, USA

**Keywords:** case report, narcolepsy, cataplexy, orexin/hypocretin, sleep disorder, physics, multiple sleep latency test, nocturnal polysomnogram, sodium oxybate, excessive daytime sleepiness

## Abstract

Narcolepsy is a chronic genetic and sleep disorder that is caused by a protein deficiency. This may affect the patients' sleep architecture and the brain's ability to control circadian rhythms. Individuals with this condition feel rested after waking but then feel tired and sleepy as the day progresses. Typical onset is during adolescence, but there is often a significant delay in diagnosis, which may markedly hinder an individual's quality of life. This case provides an opportunity to shed light on this often underdiagnosed or misdiagnosed condition by presenting a case of a remarkable individual who has persevered to be successful with the help of a timely diagnosis and aggressive off-label therapy.

## Introduction

Narcolepsy presents with excessive daytime sleepiness, and other diagnostic signs and symptoms may include sleep attacks, hallucinations, fragmented sleep, and sleep paralysis. Although clinical presentation may vary from person to person, its impact on an individual is similar across the board [1]. Patients may experience social stigma, struggle with education, and problems maintaining employment or productivity [2-3]. Evidence suggests that one out of 2000 individuals worldwide are affected with this condition [4-5]. Although it is a multifactorial condition, loss of hypocretin (orexin) is prominently associated with the genesis of narcolepsy. Data suggest that a deficit of hypocretin can be due to autoimmune disorders, family history, and injuries to the brain. Narcolepsy is more likely to develop in the spring and early summer as the winter comes to an end. Research suggests that the individuals with this condition present with high levels of antistreptolysin O antibodies, suggesting a link between the weather and narcolepsy. Furthermore, the H1N1 flu epidemic in 2009 increased the number of new narcolepsy cases and the data points toward the cross-reactivity of influenza antibodies with hypocretin receptor 2 [6-7]. This case highlights the importance of continued education and research to reduce the risk of delay in diagnosis or misdiagnosis.

## Case presentation

A 13-year-old female visited a physician in January 2007 for the evaluation of passing out associated with laughing. According to the records, the patient underwent a magnetic resonance imaging (MRI) of the brain with and without contrast, along with an electroencephalogram (EEG), in October 2007 for an evaluation pertaining to a chronic headache and generalized weakness resulting in syncopal episodes triggered by laughter. The results of these diagnostic studies were unremarkable. The patient was seen by a neurologist on September 26, 2007, for persistent symptoms of losing muscle tone triggered by a strong emotional response. The neurologist suggested that the patient should undergo a magnetic resonance angiogram (MRA) of carotid arteries because the symptom of “laughter leading to loss of muscle tone, resulting in the patient losing control and falling” may suggest syncope of a cardiovascular etiology. Furthermore, neurologist records suggest that the patient experienced four episodes of laughter leading to loss of muscle tone while she was at Disneyland, a week later. Lastly, the neurologist concluded his consultation by suggesting that no further workup was needed. Subsequently, the patient underwent an echocardiogram on October 1, 2007, to investigate her continued “syncopal episodes.” According to records, the cardiologist felt that the patient might have vasovagal syncope thus requiring an MRA and MRI of the neck followed by a Holter monitor. The results were all within normal limits as reported by the patient and her mother. Although the cardio-neuro workup was inconclusive, the patient continued to have chronic symptoms and, therefore, sought initial consultation with a sleep specialist.

The patient was seen by our practice on January 11, 2008, and was referred for a nocturnal polysomnogram (NPSG) and a multiple sleep latency test (MSLT) to investigate a probable diagnosis of narcolepsy due to exhibited signs of narcolepsy with cataplexy, sleep paralysis, excessive daytime sleepiness and hypnagogic hallucinations. The PSG results suggested a total sleep of 436.7 minutes with a sleep efficiency of 93 percent (n=89%). Moreover, rapid eye movement (REM) and sleep latencies were 94.5 minutes (n=136-156) and 10 minutes, respectively. The PSG revealed that the patient experienced one obstructive apnea lasting for 11 seconds, five central apneas, and 11 hypopneas with an apnea/hypopnea index of 2.3, which is normal. The patient verbalized not being able to move or talk during sleeping. The patient underwent an MSLT study in February 2008, which revealed a mean sleep latency (MSL) of 4.3 minutes on three 20-minute naps. The diagnostic criterion requires an MSL of ≤8 minutes and ≥2 sleep onset REM periods (SOREMPs) on MSLT. Since our patient satisfied the MSL and SOREMP components on three naps, continuing the study was not needed. Records indicate that the patient had an average sleep lasting 15 minutes, REM sleep lasting five minutes, and eight minutes average latency to REM sleep on the MSLT. A normal REM cycle occurs every 90 minutes after sleep onset, the first REM period lasts 10 minutes with each recurring REM stage lengthening. In addition to the sleep study, laboratory workup for HLA-DR15 and DQ0602 was positive, thus further supporting the diagnosis of narcolepsy.

As the patient was undergoing further studies to confirm her diagnosis, her condition and symptoms became common knowledge among her peers, which led her to be ostracized by fellow students. The situation escalated quickly to the point of feeling like she was bullied on a daily basis. Ultimately, she switched school, which added emotional stress to her life.

## Discussion

Narcolepsy is a chronic neurodegenerative condition impacting individuals physically, emotionally, and mentally. Although clinical manifestations may vary, its impact on the medical and economic burden is similar. Individuals presenting with symptoms often struggle in being diagnosed promptly, which further reduces their quality of life and functionality. Individuals with these symptoms experience negative effects on health, socioeconomic components, education, and day-to-day functionality. This can lead to a loss of jobs, further exacerbating their situation [8]. A lack of recognition of the signs and symptoms of this condition is the biggest culprit of the increasing socioeconomic burden of this condition [9].

We presented a 13-year-old patient who presented with the typical symptoms associated with narcolepsy. In an attempt to make sense of her condition, she was seen by many clinicians but was repeatedly misdiagnosed. In our care, the patient was diagnosed with narcolepsy based on her clinical presentation and the results of NPSG and MSLT. Narcolepsy is managed by a combination of pharmacologic and non-pharmacologic agents [1]. The patient was treated with sodium oxybate and modafinil. Sodium oxybate was approved by the FDA for narcolepsy in patients 18 years of age and older; a decision was taken by the treating clinician to start the therapy off-label at a reduced dose for this patient.

As with most patients, a trial and error of sodium oxybate were needed to titrate up to the therapeutic dose. The patient was started on 1.5 milligram (mg) sodium oxybate to be taken twice; however, the dose was decreased to 0.75 mg due to intolerance. In addition, the patient was prescribed 200 mg modafinil to be taken in the morning and an additional dose to be taken in the afternoon if needed. Over time, her sodium oxybate dose was increased to 4 grams taken once at bedtime. Typically, in adults, sodium oxybate is started at 2.25 grams (g) at bedtime and repeated three to four hours later due to its short half-life for a total of 9 g. Most titrations take several weeks to months at times. Along with medication, non-pharmacologic interventions, such as not being able to swim, not being able to enjoy riding a bicycle with her friends, requesting access to medication at all times, and needing extra time for exams, along with many other interventions, were instituted. Basically, she had to avoid all activities where laughter may induce cataplexy, which may increase the risk of bodily harm.

In a recent visit, the patient stated that she is tolerating her sodium oxybate and modafinil at a dose of 4 grams taken once per night without any complications and that her condition has stabilized. Timely management of her condition has improved her day-to-day functionality along with improving her quality of life. She stated that a combination of pharmacologic and nonpharmacologic interventions have allowed her to be normal and had the confidence to pursue her aspirations. Today, she is a Physics professor at a local institute and a role model for other individuals with narcolepsy-like symptoms. In fact, the patient claims to have recognized at least two other students with symptoms associated with narcolepsy but without an official diagnosis.

## Conclusions

Narcolepsy is a rare condition that significantly impacts patients and their families. Although clinical manifestations may vary, the presence of excessive daytime sleepiness is one of the most common symptoms and should point towards the possibility of narcolepsy as a diagnosis. The workup and management of this condition may be simplified if the clinician gains familiarity and experience treating similar patients. It has been tremendously rewarding to see patients with a significantly improved quality of life post-treatment. Therefore, this case is an excellent example illustrating the need for continued research and education to reduce the delay in the diagnosis of this condition, especially because existing management is proven effective in reducing symptoms and improving quality of life. Our patient suffered a major social set back by having to switch schools and being essentially bullied by other students. If the diagnosis of narcolepsy was never made, it is highly likely the patient would have gone on to experience unnecessary social, economic, and personal obstacles or challenges. Early identification of narcolepsy is key to prevent extensive testing and a delay in treatment. Once this patient was diagnosed, she was not only offered pharmacologic management but support group help and other non-pharmacologic management strategies, including sleep hygiene recommendations and scheduled naps, which are typically very refreshing for the patient. Ultimately, the patient has become very familiar with the signs and symptoms of her condition and has even helped other peers with narcolepsy from a patient education and support standpoint.
